# DNA evidence for global dispersal and probable endemicity of protozoa

**DOI:** 10.1186/1471-2148-7-162

**Published:** 2007-09-13

**Authors:** David Bass, Thomas A Richards, Lena Matthai, Victoria Marsh, Thomas Cavalier-Smith

**Affiliations:** 1 Department of Zoology, The University of Oxford, South Parks Road, Oxford, OX1 3PS, UK; 2 Department of Zoology, The Natural History Museum, Cromwell Road, London, SW7 5BD, UK; 3 School of Biosciences, Geoffrey Pope Building, University of Exeter, Streatham Campus Exeter, EX4 4QD, UK

## Abstract

**Background:**

It is much debated whether microbes are easily dispersed globally or whether they, like many macro-organisms, have historical biogeographies. The ubiquitous dispersal hypothesis states that microbes are so numerous and so easily dispersed worldwide that all should be globally distributed and found wherever growing conditions suit them. This has been broadly upheld for protists (microbial eukaryotes) by most morphological and some molecular analyses. However, morphology and most previously used evolutionary markers evolve too slowly to test this important hypothesis adequately.

**Results:**

Here we use a fast-evolving marker (ITS1 rDNA) to map global diversity and distribution of three different clades of cercomonad Protozoa (*Eocercomonas *and *Paracercomonas*: phylum Cercozoa) by sequencing multiple environmental gene libraries constructed from 47–80 globally-dispersed samples per group. Even with this enhanced resolution, identical ITS sequences (ITS-types) were retrieved from widely separated sites and on all continents for several genotypes, implying relatively rapid global dispersal. Some identical ITS-types were even recovered from both marine and non-marine samples, habitats that generally harbour significantly different protist communities. Conversely, other ITS-types had either patchy or restricted distributions.

**Conclusion:**

Our results strongly suggest that geographic dispersal in macro-organisms and microbes is not fundamentally different: some taxa show restricted and/or patchy distributions while others are clearly cosmopolitan. These results are concordant with the 'moderate endemicity model' of microbial biogeography. Rare or continentally endemic microbes may be ecologically significant and potentially of conservational concern. We also demonstrate that strains with identical 18S but different ITS1 rDNA sequences can differ significantly in terms of morphological and important physiological characteristics, providing strong additional support for global protist biodiversity being significantly higher than previously thought.

## Background

In 1934, Baas-Becking stated with respect to microbes that 'everything is everywhere' with the important qualification 'the environment selects' [1]. This ubiquitous dispersal hypothesis (UDH) of global dispersal with transient local occurrence is the focus of increasingly active debate [2-5]. It is supported by many surveys of protist morphospecies on a global scale, but exceptions have been proposed [6-8]. Microbial cosmopolitanism is thought to be driven primarily by the random dispersal pressure generated by vast population sizes of cells below a body-size of approximately 1 mm [9]. A dependent hypothesis states that global protist species richness is consequently relatively low, as opportunities for allopatric divergence are limited or absent, and that the local to global species ratio is high, theoretically approaching 1 [5]. In other words, the diversity of microbial species found in one locality generally represents global levels of diversity within that habitat. However, recent molecular studies have contributed several new perspectives [7,10-16], mainly 1) that diversity of 18S rDNA genotypes (18S-types) within morphospecies can be very high; 2) that many 18S-types have cosmopolitan distributions, and 3) some 18S-types within morphospecies have ecologically and/or geographically restricted distributions. These and other investigations indicate that protist morphospecies usually offer too crude an evolutionary resolution to be ecologically informative [17-21], reinvigorating the debate about at what level of taxonomic distinction are protists in fact cosmopolitan [22]. Foissner and colleagues promote a moderate endemicity model [23,24], which proposes that while some protist morphospecies have cosmopolitan distributions, other, perhaps rarer protists have restricted distributions. Evidence for this hypothesis is comprehensively reviewed in [25]. Our culture-independent, molecular screening approach provides a robust framework for testing the moderate endemicity model at high phylogenetic resolution, as it does not distinguish between protists which differ in ease of propagation in the laboratory, or ease of detection and study by microscopy.

**Figure 1:**
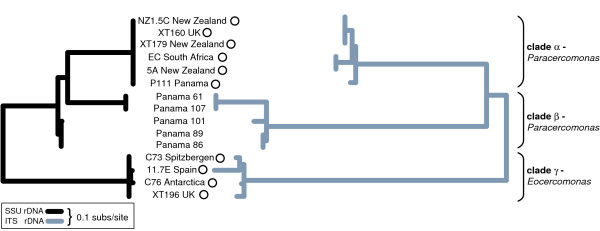
**Comparisons of evolutionary changes in the 18S and the ITS1 sequence**. Left tree (black): Maximum likelihood (ML) phylogeny of cercomonad 18S rDNA sequences calculated from an alignment of 15 sequences and 213 nucleotide positions from the hypervariable V4 region. Right tree (grey): ML phylogeny of corresponding ITS1 rDNA (213 positions). Branch lengths and scale bars standardised to illustrate the much greater evolutionary rate of ITS1 than even the most hypervariable 18S rDNA region. Equivalent parsimony analyses using gaps as a 5th character (not shown) showed a parsimony score of 367 (minimum changes) for ITS1 and 120 for the V4 18S rDNA region. Cultures used for ecological and phenotypic character experiments (Table 2) marked by circles.

**Figure 2:**
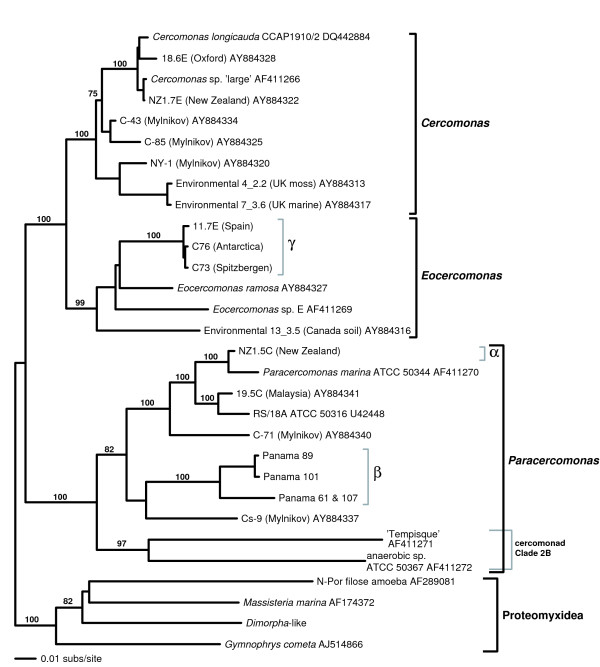
**Phylogeny of cercomonads**. BioNJ bootstrap tree (1000 replicates; corrected for Γ & I) of a subset of known cercomonad lineages, representing all known main cercomonad clades and genera (*Cercomonas*, *Eocercomonas*, and *Paracercomonas*) as defined by Karpov et al. [14]. The composition and phylogenetic position of the groups α, β, and γ forming the basis of this study are shown. A group-specific primer set was designed for each of these groups (see Methods).

**Figure 3:**
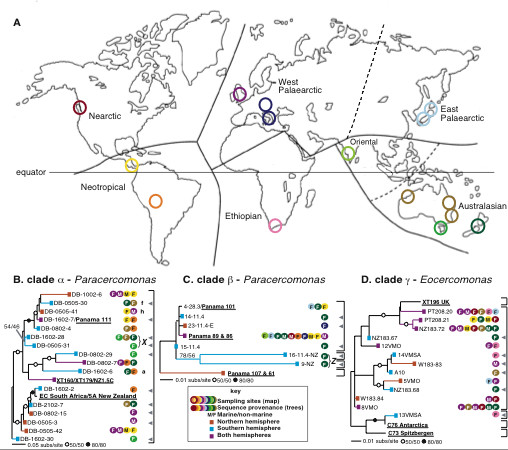
**Global provenance, biogeography and phylogeny of cercomonads**. (**A**) World map showing major animal biogeographical regions. (**B-D**) 18S-ITS1 rDNA phylogenies of groups α (278 nucleotide positions), β (414 positions), and γ (335 positions). See Methods for details of each sequence dataset. Coloured discs correspond to hoops on map. Grey arrowheads indicate sequences found in only one biogeographical region. Square black brackets indicate 18S-genotype boundaries. *X *and *Z *(bracketed) indicates a possible endemic genotypic radiation. Branches marked a, f, and h in (**B**) are discussed in relation to additional sampling and ITS-type-specific primer probing. Branches without coloured discs originate from cultures. Sampling range differs among the three groups (Table 1). Key to bootstraps (ML/parsimony) given to right of scale. Bootstraps shown only when equal to or exceeding the value stated, although precise values are shown for clades marked *X *and *Z*, as these are discussed in the text as potentially geographically-restricted clades. Branch labels underlined and in bold refer to culture-derived sequences, including those used in the culture experiments. NB: in some cases a single branch corresponds to >1 culture or both environmental and culture-derived sequences. The long branch for 11.7E is not shown in (**D**) for the sake of clarity. It was not detected in any environmental library.

Cercomonads are benthic heterotrophic flagellates (5–50 μm long) comprising the genera *Cercomonas*, *Eocercomonas*, and *Paracercomonas *[14]. They are abundant in soils [26-28] and aquatic environments, being among the most prominent bacterivorous protozoa. As global dispersal should theoretically be easier for marine protists, making endemicity less likely, we preferentially sampled soil and freshwater environments to test the UDH more rigorously. As some cercomonad 18S-types are globally distributed [10,14], we used ITS1 rDNA sequences, which evolve significantly faster than 18S rDNA sequence (Fig. 1), to determine whether geographically restricted distributions could be detected within cercomonad 18S-types at this higher level of resolution. As there are hundreds of different cercomonad 18S-types [14], we used PCR primers targeted to three very narrow taxonomic groups comprising 1 to 3 18S-types each (Figs 2, 3). This approach enabled us to maximize the chances of detecting the same genotypes in different samples even when rare. To be representative, we targeted three different regions of the cercomonad tree incorporating at least one genus from each of the two main cercomonad clades (Fig. 2) [14]. Our intensive sampling of the rapidly-evolving ITS1 rDNA marker reveals both ubiquitous and restricted distributions and thus demonstrates that biogeography in microbes and macro-organisms differs only in degree. We also show that cercomonad strains within a single 18S-type can differ in terms of phenotype and ecological preferences.

## Results and discussion

Fig. 2 is a general tree of cercomonad diversity, showing the positions of the three very narrow lineages targeted in this study. In order to achieve a very intense sampling of these lineages and avoid amplifying a vast number of intermediate lineages (much greater than shown on this sample tree) irrelevant to our present purpose we used PCR primer pairs including one primer highly specific for each narrow lineage (see Methods). Fig. 3 shows that despite the extreme narrowness of our primer specificity we recovered up to 18 distinct ITS-types within a single 18S-type, and that two groups (β,γ – Fig. 3C,D) have some globally distributed ITS-types (six found in both hemispheres and three or more biogeographical regions, treating the Palaearctic as a single region). The other group (α – Fig. 3B) was sampled from only three biogeographical regions (Table 1), and revealed many ITS-types from two regions but only one from all three. Most ITS-types (21 of 36) were detected only in one region. The provenances of all 18S- and ITS-types are shown in Table 1. As more libraries were screened within each clade, individual ITS-types were increasingly recovered from more than one continent. This suggested that the true diversity present in the environment remained undersampled. To explore this further, we did two things.

**Table 1:** Sampling and provenance of genotypes sampled

**Biogeog. region**	**Provenance**	**Habitat**	**#eDNAs screened (# successful amplifications, where known)**	**#18S-types**	**#ITS-types**	**#ITS-types found only in this biogeog. region**	**Date of collection (MM.YY)**
**GROUP α**							

Australasian	New Zealand	Soil & f/w sediment	8 (4)	1	4	6	02.03
	Australia	Soil	4 (1)	1	4		12.04
	Tasmania	Soil	4 (1)	1	4		12.04
Neotropical	Peru	Soil	7 (3)	1	5	2	07.05
	Panama	Freshwater sediment	4 (4)	1	4		03.03
	Panama	Coastal sediment	5 (3)	1	2		03.03
W. Palaearctic	Oxfordshire	Soil	7 (4)	1	4	2	07.03
	UK	Coastal sediment	8 (7)	1	4		05.02

**GROUP β**							

Australasian	New Zealand	Soil & f/w sediment	8	3	6	4	02.03
		Coastal sediment	13	1	1		02.03
	Tasmania	Soil	4 (1)	1	1		12.04
Oriental	Calcutta	Soil	4 (3)	1	1	0	02.05
Neotropical	Panama	Freshwater sediment	7	1	2	0	03.03
	Panama	Coastal sediment	8	1	1		03.03
	Peru	Soil	12	1	1		07.05
W. Palaearctic	UK & Europe	Coastal sediment	6	1	1	1	'03 & '04
	UK & Europe	Soil	7	1	1		'03 & '04
E. Palaearctic	Japan	Freshwater sediment	6 (5)	1	2	0	12.04
Nearctic	BC	Coastal sediment	5	1	1	0	06&09.02

**GROUP γ**							

Australasian	New Zealand	Soil & f/w sediment	8	1	5	3	02.03
		Coastal sediment	5	1	2		02.03
	Australia	Soil	4	1	4		12.04
Neotropical	Panama	Soil & f/w sediment	4	(1^1^)	(1^1^)	(1^1^)	03.03
	Panama	Coastal sediment	7	1	2		03.03
Ethiopian	South Africa	Soil	11	2 (+2^1^)	4 (+2^1^)	2 (+2^1^)	12.03
W. Palaearctic	UK	Coastal sediment	11	1	5	1	05.02
	Oxfordshire	Soil	4	1	5		07.03
E. Palaearctic	Japan	Freshwater sediment	5	1	2	0	12.04
Nearctic	BC	Soil	6	1	5	0	06&09.02

(eDNA = environmental DNA sample – see Methods for more details.)

^(1) ^These additional genotypes were also detected, and were not recovered from any other site. (Not shown on Fig. 3.)

Firstly, for group α, which contains the largest number of putatively geographically restricted ITS-types, more clones were sequenced from the libraries constructed from the largest number of successfully amplified eDNAs (environmental DNA samples; see Methods) – those from Europe marine and Panama marine – and those producing the highest number of putatively 'endemic' (and incidentally the most divergent) ITS-types – New Zealand and Australia; see Table 1. All 44 new sequences from these four libraries matched ITS-types previously recovered; suggesting that sampling of these libraries was at or near saturation (16 ITS-types being recovered from 110 sequence reads). One of these ITS-types (h, Fig. 3B), previously only recovered from Panama, was thereby shown to be present in European samples. Secondly, primers specific to subclades of α (Fig. 3B) were designed in order to probe intensively previously untested eDNAs to determine whether ITS types retrieved from only one or two locations in the original analysis were in fact more widely distributed. PCR screening and sequencing showed that clade a (Fig. 3B), previously detected only from Peru was also present in New Zealand, although clade f, originally from Australia/New Zealand could still only be found in Australasian samples. These additional experiments show that there was at least some undersampling in our initial data set. Thus some ITS-types are probably actually more widely distributed than Figs 3B–D suggests, especially for groups β and γ where this more intensive additional sampling was not done. However, this extra sampling of group α increased the geographic distribution for only two clades.

**Figure 4:**
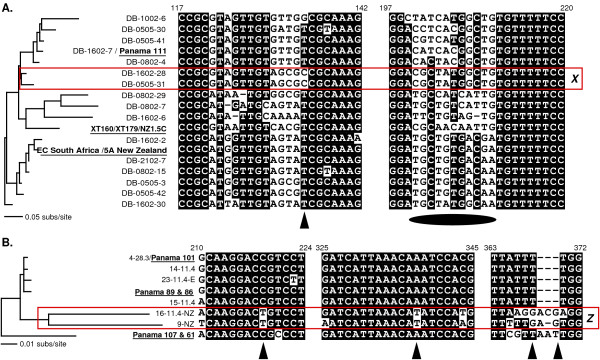
**Shared alignment characters supporting putative geographically restricted clades *X *and *Z *(boxed in red)**. (**A**) *Paracercomonas *group α tree; (**B**) *Paracercomonas *group β tree (taken from Fig. 3). Characters at specific alignment positions are coloured black when 0.8 of positions agree; otherwise they are unshaded. Unique clade-specific alignment characters are illustrated with triangles across the bottom of the alignments. Shared sequence motifs in a hyper-variable section are illustrated with an oval across the bottom. Sequence position numbers across the top refer to DB-1002-6 and P101 PANAMA.

Furthermore, of 21 ITS-types detected from only one biogeographical region (grey arrowheads, Fig. 3B–D), five were retrieved from more than one library, all within group α. Given the high level of our sampling, this suggests that these ITS-types are biogeographically restricted (our additional sampling and lineage-specific primers found none of these ITS-types elsewhere). It is particularly striking that sequence DB-0505-31 on Fig. 3B was detected in three separate freshwater Australasian libraries and in no others. One should therefore not extrapolate from our finding four clear examples of global ubiquity to the conclusion that this is true of all cercomonad ITS-types. It cannot reasonably be argued that all genotypes are both globally distributed and equally abundant on all continents. If they were, we should easily have found DB-0505-31 in libraries constructed from other continents. It seems probable that it is either really restricted to Australasia or else dramatically more abundant there at the time of sampling. Even the latter is contrary to the core assumptions of the UDH, unless it could be demonstrated that none of the samples on any other continents were from ecologically similar sites to those sampled from Australasia. It is possible that such ITS-types have a patchier distribution than those that are easily found at many sites globally; but although patchiness combined with rarity and undersampling might account for the restricted distribution of some of the sequences we encountered in single libraries, it is unlikely to explain all examples of apparent biogeographic restriction. Three of the five ITS-types detected in more than one library but from only one biogeographical region were from Australasia. These are shown in Figs 3B and 3C as clusters *X *(including DB-0505-31 mentioned above) and *Z*. Both putative clades are supported by multiple unique and identical sequence motifs in conserved alignment regions of ITS1, as shown in Fig. 4. *X *and *Z *are illustrative of the dominance of Australasian-derived sequences (12 ITS-types) in the set of 21 ITS-types recovered from only one biogeographical region (= 57%). As is well known, Australasia is the most geographically isolated continent and shows exceptionally high endemicity for higher animals, e.g. monotremes and marsupials, and plants [29,30]. Perhaps it also has an unusually high level of endemic cercomonad genotypes.

It is theoretically possible that seasonal differences in cercomonad strain occurrence and/or relative abundance could account for some of the distribution patterns described here. However, unpublished data from comprehensive sampling of cercomonads at several sites in the UK suggest that if such shifts in abundance occur they would certainly not preclude detection by the very specific primers used in this study (Howe and Bass, unpublished). Many, if not all, genotypes detected at these sites were present in both winter and summer. The times of sample collection for the present study are given in Table 1.

Nine ITS-types were recovered from both marine and non-marine environments (Fig. 3B–D). Though only a small proportion of the total, this suggests that the marine/non-marine transition is unusually easy and rapid for these cercomonads. Protozoa in marine and non-marine habitats are largely distinct [11,12]. Many major taxa are restricted to one or the other environment: Radiozoa, Phaeodarea, Xenophyophorea, Pelagophyceae, and most foraminiferan subgroups are exclusively marine, whereas Mycetozoa (for example) are never marine. Even in groups where some marine/freshwater shifts have occurred, there are many substantial subclades that are apparently exclusively one or the other, for example in bodonids and *Goniomonas *[11,12,15]. Some lineages have clearly undergone such shifts more frequently than others and are apparently more adaptable in this respect. Yet even in such groups not all strains can be encouraged to adapt physiologically from 'marine' to 'non-marine' (or *vice versa*) conditions in laboratory cultures, and even where this can be achieved there is no guarantee that it can and does happen in nature. On present evidence the fraction of protist lineages able to live in both marine and non-marine habitats is very small.

The unusual capacity of some cercomonads to make relatively rapid switches between marine and non-marine environments might greatly facilitate their global dispersal. To see if an ability to grow in marine habitats is a general property of these cercomonads, we tested strains from clades α and γ isolated from soil or freshwater in marine media: strains XT179, NZ1.5C, XT160, EC South Africa, 5A New Zealand, and Panama 111 (all clade α), and C73, XT196, 11.7E, and C76 (clade γ) were inoculated into media at different concentrations, and observed for five weeks. The results are summarized in Table 2. None of the strains could grow in full strength seawater medium (SWM), showing that not all strains with the same 18S rDNA sequence can grow in both marine and non-marine media, even though some ITS genotypes can be found in both habitats (Fig. 3). In fact, only strain 11.7E grew at all well in 50% SWM, although the cells were unusually inactive. All other strains tested grew very poorly or not at all in 50% SWM, and grew with highly varying degrees of vigour in 25% SWM. Our observations, based on replicated, standardized cultures, show that cercomonad strains with identical 18S rDNA but different ITS1 sequences can differ in several respects: salinity tolerance, propensity to form cysts, vigour in standard non-saline media (growth density and persistence over time), size and basic cell morphology (Table 2). In our experiments the greatest differences in salinity tolerance were between strains with slightly different 18S sequences, even when the degree of genetic difference was at or below our threshold for defining unique 18S-types. This reinforces the growing awareness that taxonomic units based on 18S rDNA identity are at least sometimes insufficiently resolving to identify distinct ecological entities [18-21]. Given the very close rDNA similarity between the strains studied, it appears that the evolution of many physiologically and evolutionarily significant characters may be relatively rapid in cercomonads.

**Table 2:** Results of culture experiments: ability of clonal cercomonad strains to grow in various marine media and other strain characteristics

**Clade^a^**	Strain	**18S identity^b^**	**ITS1 identity^b^**	**Morph. identity^c^**	**Salinity tolerance^d^**	**Size (μm)^e^**	**Cysts^f^**	**Vigour^g^**
**α**	XT179	1	1	1	() (motile cells)	5–7		
α	NZ1.5C	1	1	1		5–6		
α	XT160	1	1	1		5–7		
α	EC	1 *	2 *	1	(immobile cells)	5–7	X?	
α	5A	1	2 *	2	N/A	6–8	X?	
α	P111	1	3	N/A	N/A	5–7	X	
α	SCCAP C1^h^	1	N/A	N/A	N/A	4–10^h^	()^h^	N/A

γ	C73	1	1	N/A		4–6	X	
γ	XT196	1	2	N/A	N/A	5–10	N/A	
γ	C76	2	3	N/A	X	5	X	
γ	11.7E	3	4	N/A		5–7		

N/A = no data

* Although the ITS1 sequences for EC and 5A are identical, and 18S rDNA is identical in the V4 region (on which Fig. 1 is based), EC has an insert near the 3' end of 18S and is therefore clearly a different strain. This is the only strain in this study known to have such an insert.

^a ^see Figs 1, 2, and 3.

^b ^Identical numbers indicate strains with identical sequences.

^c ^Identical numbers indicate strains with strain traits other than size (flagella length:body length, form and frequency of plasmodium formation, propensity to form cell clusters, etc.) which were indistinguishable from others in the same medium under light microscopy (200× phase contrast).

^d ^Overall performance of strains under a range of saline medium regimens, as described in Methods. X means that no living cells were seen.

^e ^Size range determined from 3+ independent cultures observed over a period of 18 months.

^f ^Indicates the propensity of the strain to form cysts in Volvic & grain cultures, summarized from observations of 3+ independent cultures observed over a period of 18 months. X = no cysts seen,  = cysts infrequent and sparse,  = cysts regular at low to medium densities,  = cysts readily formed and seen at high densities.

^g ^A measure of the ability of the strain to form dense cultures in Volvic & grain medium, which persist well over time (2+ months in unmanipulated cultures), summarized from observations of 3+ independent cultures observed over a period of 18 months.  = rarely or never reach high density,  = may reach medium to high density but do not persist,  = usually reach high densities and/or persist for long periods without encysting or disappearing from the culture.

^h ^See ref. 31. Not included on Figs 1 and 3 as there is no ITS1 sequence available. Not included in culture experiments.

The strains used for the culture experiments were all isolated from soil and freshwater and we had no prior evidence suggesting that any could grow in marine media; none of these cultured ITS-types correspond to those retrieved from the libraries (also indicative of non-exhaustive sampling of ITS-types). The culture experiments suggest that some ITS-types are tolerant to a wider range of salinity levels than others, and that there are ITS-types which are genuinely restricted to non-marine environments. Of the 19 ITS-types detected in more than one library, none was exclusively marine, whereas nine were found only in non-marine libraries. This suggests a freshwater ancestry for these cercomonad groups and that only a minority of ITS-types tolerate high salinity. Many protists persist in unfavourable environments by encysting. Different ITS-types may also differ in propensity to form cysts; for example in *Paracercomonas ekelundi *(SCCAP_C1 – Table 2) they are 'formed readily' [31], whereas this is not true of all other ITS-types within that 18S-type (Table 2). Our culture experiments also showed that cultures of identical ITS-types were more similar to each other than those with different ITS1 sequences in respect of a suite of traits, which are likely to be ecologically significant (Table 2).

These observations suggest that differences in ITS sequence may be useful as markers for identifying clearly distinct biological/taxonomic units. It has been shown for strains from a broad range of taxa (ciliates, green algae, plants, sea urchins, and abalone) that boundaries of sexual compatibility appear to coincide with phylogenetic groups defined by individual compensatory base changes in ITS2 rDNA [32,33]. More recently, Amato et al. [34] have shown that cryptic sister species are profuse in the diatom genus *Pseudo-nitzschia*, where biological species correspond to strains with identical helix regions of ITS2. In *Pseudo-nitzschia *the degree of differentiation between strains was similar for ITS2 and ITS1, although in at least one case divergence in ITS1 sequences was more marked than that in ITS2 [34]. With this one exception, ITS1 sequences within a biological species were identical. If cercomonads are similar to diatoms, ciliates, and green algae in this respect, then the majority of 'species' would have no detectable variation in ITS1, and the majority of separate ITS1 types would be separate species. In this scenario it would be unsurprising that distinct ITS1-types respond differently to culture variations. However, it is not known whether cercomonads are sexual, and there are insufficient morphological characters (without using electron microscopy) that could be robustly related to ITS divergence. Thus biological species cannot currently be defined for cercomonads.

## Conclusion

Our study is so far unique in screening such large numbers of globally distributed eDNAs with PCR primers so narrowly targeted as likely to include relatively few 'sibling species'. Our data demonstrate on the one hand that global dispersal of at least some cercomonad ITS-types occurs faster than divergences among these relatively fast-evolving markers. On the other hand, many ITS-types appear to be more restricted in distribution; several are candidates for being genuinely endemic to one biogeographic region. Still more intensive sampling would be necessary to decide whether the majority of strains found only once in this study fall in the cosmopolitan or endemic category. However, our results are more decisive than earlier indications in the ciliate *Paramecium *that one sibling species of the *P. aurelia *complex may be endemic to Eurasia [35]), whereas many are cosmopolitan. Some assertions of endemicity in *Paramecium *[35] were attributable to undersampling [36]. Several species are almost certainly genuinely geographically restricted (i.e. not cosmopolitan; [35,37]), though in some cases apparently because of latitudinal/climatic factors [35] rather than limited dispersibility, and thus not counter-examples to the ubiquitous dispersal theory. Targeted species-specific environmental DNA sampling is needed in these ciliates along the lines of our present study to distinguish between undersampling, latitudinal/climatic geographic restriction, and genuine historically based endemicity. Thus, just as for animals and plants, some protist species or reasonable surrogate markers for species such as ITS genotypes are genuinely cosmopolitan, whereas others show continental endemism. In this respect our results support the moderate endemicity model described above.

To measure true levels of endemism in microbial eukaryotes would require extensive testing of a large number of protist groups from across the eukaryote tree with at least as much intensity as we demonstrate in this paper. What is now clear is that some of the perception of greater cosmopolitanism in microbes [5] is attributable to the taxonomic artefact of lumping huge numbers of genetically very different organisms into a single crude 'morphospecies'. Our study confirms that the 18S-type, currently the most frequently used marker for distinguishing protist strains, is too coarse for distinguishing between ecologically and biogeographically significant units in cercomonads. The next key question is whether cercomonad ITS-types correspond to biological species. If they do, cercomonad species numbers are likely to be at least 100 times greater than yet described. Thus currently the number of protozoan species is probably hugely underestimated.

## Methods

### Environmental Sampling

Globally dispersed environmental samples were collected from soil, freshwater, and marine sediments, by different individuals using separate collecting apparatus, and DNA extracted under sterile conditions as described previously [10]. Provenances of all environmental DNA extractions (eDNAs) used in this study are shown in Table 1. In each sampling region a range of habitats was sampled over as broad a geographical area as possible. A broad overview of sites is as follows. Panama: Pacific and Caribbean slopes (coast and interior); New Zealand: North and South Island (coast and interior); Peru: soils from both sides of the Andes – predominantly lowland wet tropical forest; Tasmania: soil samples from around the perimeter of the island; British Columbia: a transect from Vancouver to the Okanagan valley, plus the West coast of Vancouver Island; Europe: primarily UK but also France, Germany, and Greece. Less widespread samples were taken in India, Japan, and Australia. The habitats sampled included wet and dry soils, scrapings from rocks, sediments and decaying material associated with mosses, freshwater sediments and scrapings from submerged rocks, plants, and detritus, and coastal sediments and rock scrapings.

### Molecular Screening

Cercomonad clade-specific primers were designed from culture-derived 18S rDNA alignments: forward primers VM1B (group α) 5'-TAGTACAACGTAACCCTTGGT, VM4B (group β) 5'-AATAGTGGGGCTTAGGCTTGCC and VM5 (group γ) 5'-CTTAGTGAGCTTCAGAGATTGATGTACATA. Reverse primers, situated in 5.8S rDNA, were the eukaryote-universal 369R 5'-TCGCATTACGTATCGCATTTCGCTG (first round PCR) and Bii 5'-TGCGTTCTTCATCGWTACGAG (nested PCR). The primers were designed from a total cercomonad alignment of approximately 150 sequences to be specific to one or more cercomonad 18S-types. The phylogenetic positions of the three groups is shown in Fig. 2 in the context of a subset of all cercomonad lineages. The exact location of the primer site in the SSU was determined by the existence of regions that provided the degree of specificity required. This degree of specificity required between groups: α comprises a single 18S-type because it was relatively frequently represented in our database, and therefore we judged that it would be relatively easy to find in eDNAs. β comprises three, quite divergent, 18S-types – the interest here was that we had only previously retrieved members of this clade as cultures from Panama. γ comprises three very closely related 18S-types, individually for which it would have been effectively impossible to design reliably robust primers. All primer sets were tested with known positives and negatives, and a PCR protocol chosen that amplified only sequences within those clades.

Nesting was necessary as the initial amplifications frequently produced faint/invisible bands after gel electrophoresis. PCR cycling conditions were 5 min. initial denaturation (95°C), followed by 35 cycles of 32s at 95°C, 36s at 68°C (α), 65°C (β and γ), 1.5 min. at 72°C, then 5 min. at 72°C. Multiple PCR products for each environment were pooled prior to separation on a 1% agarose gel from which they were excised and cleaned using the GFX Gel Purification Kit (GE Healthcare). The resulting fragments were cloned using the TOPO TA Cloning Kit (Invitrogen). At least 16 colonies were selected from each library and sequenced as previously reported [10].

Sequences of each group were aligned manually using SE-AL [38], and preliminary BioNJ trees constructed [39]. These were used to refine the environmental sequence alignment and reduce the environmental sequence sampling (113 down to 18 group α sequences; 110 to 9 group β; 109 to 16 group γ) and determine genotype boundaries as described below. All effectively identical sequence copies were then removed from the alignment, leaving only those sequences deemed to be clearly different from each other. The length of the amplicon for each group was dependent on the position of the group-specific (forward) primers in the 18S rDNA, at the 5' end. The full amplicon length was retained in the alignments for Figs 3B–D in order to maximize tree quality. The alignment lengths analyzed were as follows: group α = 278 positions; group β = 414 positions; group γ = 335 positions. Final trees were calculated using maximum likelihood (ML) with an eight category Γ + I site rate variation model. All model ML parameters were previously estimated using likelihood treescore from a preliminary BIONJ tree [39,40]. We used a general time-reversible rate matrix (GTR) with six substitution categories estimated from the alignment using the BioNJ tree. Additional analyses was performed by maximum parsimony with gaps coded as a 5^th ^character state [39]. In both approaches 10 random replicate heuristic searches with stepwise addition and TBR were used for the topology searches (ML trees shown on Fig. 3). 100/1000 bootstrap replicates (for ML/parsimony respectively) were conducted using the same settings.

### Evolutionary rate comparison (Fig. 1)

To confirm that the cercomomad ITS sequences evolve significantly faster than any region of 18S rDNA sequence we constructed complementary alignments for both datasets in SE-AL using all available sequences derived from cultured representatives of the three cercomonad groups studied. We calculated the phylogenies using parsimony and ML as described above [39,40]. Scales of the ML results were standardised and differences in parsimony scored (minimum changes) for each alignment were recorded. The length of both 18S and ITS1 alignments was 213 positions. The beginning of ITS1 was determined to follow the end of the 18S sequence as given in [41], which point coincided with the junction between conserved (18S) and highly variable (ITS1) regions in our extensive cercomonad rDNA alignment. The V4 region of 18S rDNA was selected as the most variable region of that gene, to provide the most appropriate comparison between the genes and also because it is the region we use to define unique 18S-types, in a similar way to that described for ITS1, below.

### Group α subclade-specific primers

An initial amplification was carried out with VM1B and 369R. The products of each reaction were diluted 1:10 and used in a nested PCR with VM1B and highly specific subclade primers for the two subclades a and f on Fig. 3B. These primers were situated in ITS1 rDNA to provide the necessary high degree of specificity. The PCR protocols were optimimzed in the same way as described for α-, β-, γ-specific primers. For clade a, two specific primers were used, a1 and a2, the latter being used in a second nested PCR step using the diluted products of the VM1B-a1 amplification. The corresponding primer sequences are a1: 5'-ATTATCAGTTAAAGGCTTTCGCCTAG; a2: 5'-ACAGAATCCCTCATCGCTAGGATG (clade a, originally detected only from Peru); f (clade f, originally detected only from New Zealand and Australia): 5'-AGAGAGAAGCGGGCCATAACAC. Following PCR optimization of each primer set using known positives and negatives, primer sets a and f were each used to probe eight eDNAs from four locations: Australia, New Zealand, Tasmania, and Panama, including the eDNA from which bands were obtained using the VM1B-Bii set. The PCR products from each location were pooled separately and run on a gel. Primer f produced a band only for Australasian region samples, whereas primer a2 (following nesting) produced bands for both Australia and Panama (no bands for either were seen after the first nested PCR using a1). The two visible bands were cloned and sequenced, yielding only the identical ITS-type to which they were designed. This suggests that a double nesting PCR approach (i.e. three serial amplifications in all) can be necessary when probing with such specificity, probably because of the low concentrations in environmental samples of some individual ITS types.

### Estimation of clonal variation in rDNA cistrons and PCR/sequencing error

As a control for properly interpreting whether observed differences in ITS could be caused by polymorphic differences among the many copies within a single genome and/or by PCR/sequencing errors, we made rDNA libraries from clonal cultures and measured intra-clonal differences in the resulting alignment for two phylogenetically distant cercomonad strains (*Cercomonas *sp. 'AZ6' ATCC 50418 &*Cercomonas *C59 [14]). For each strain 24 clones were sequenced on both strains along the full amplicon length. The sequence difference percentage was calculated by dividing the total number of mismatches by (24 × number of alignment positions) then multiplying by 100. The distribution of sequence variation in the alignment is shown in Table 3, which shows that sequence variation caused by combined PCR nucleotide incorporation error and intra-clonal rDNA polymorphism is low (mean = 0.1%) and that the more rapidly evolving regions of the gene are not more variable in this respect than relatively conserved regions. It is not possible to distinguish between actual intra-nuclear differences between gene copies and nucleotide misincorporation errors during the initial PCR. However, this approach estimates or overestimates the upper limit of intra-nuclear polymorphism levels and provides a measure of the total intrinsic level of errors generated by our methodology. The alignments were created from an initial PCR amplification of DNA extracted from each culture using the cercozoan-specific primer 1259F (5'-GGTCCRGACAYAGTRAGGATTGACAGATTGAAG) and the eukaryote-universal primer 28Sr1 (5'-CGGTACTTGTTCGCTATCGG). The primers used for sequencing were 1259F, 369R, pre-Bf (5'-GTAGGTGAACCTGCAGAAGGATC) and 1733R (5'-TGATCAAGTTTGATTCAGTTCTCGGAT).

**Table 3:** Sequence variation within libraries constructed from two clonal cercomonad cultures

**Strain**	**Position on rRNA cistron**	**Number of positions examined**	**Number of mismatches**	**Sequence difference (%)**	**Variability of region**
AZ6^1^	18S	545	15	0.11	Relatively conserved
C-59^1^		535	11	0.09	
AZ6	ITS1-5.8S-ITS2-28S (D1/D2 region)	603	11	0.08	Relatively variable
C-59		609	20	0.14	
AZ6	whole fragment	1148	26	0.09	(average)
C-59		1144	31	0.11	

^1 ^see ref. 14

### Definition of unique genotypes

The threshold of divergence used for defining unique genotypes was much higher than divergence due to combined errors, which were measured as described in the preceding section. The standard requirement for two genotypes to be considered distinct from one another was at least four differences within the most variable positions of their ITS1 sequences. A degree of subjectivity regarding these decisions was allowed, depending on the degree of variability of the exact region of the alignment in which they occurred. However, these decisions always erred on the side of stringency and it is likely that we have *underestimated *the total number of ITS-types detected in this study. If so, this would mean that on average, we *overestimate *the degree of cosmopolitanism of each ITS-type. Single nucleotide differences in conserved regions of the alignment were discounted as likely sequencing errors and/or polymorphic sites. One example of each unique ITS-type produced by this study has been deposited in GenBank (Accession numbers EF174336-EF174385).

### PCR recombination (chimaera formation)

Chimaeric sequences are caused by DNA template switching between PCR cycles. We took the following precautions to eliminate chimaeras from our dataset. 1) Our amplicons were as short as possible, and PCR extension times were set for at least three times as long as necessary (this was determined empirically). Chimaera formation frequency is relatively low in each experiment [10] and is minimal where amplicon length is small and extension time is long. 2) The short ITS1 region was scanned in order to check that the sequence variability and signature sequences were consistent along its length. If a PCR recombination event had occurred it would be easily detectable. 3) Identical sequences recovered from more than one library were deemed non-chimaeric. The likelihood of the exact same chimaera being produced in independent libraries on multiple occasions is extremely remote.

### Culturing

To test whether monoclonal cercomonad strains isolated from non-marine environments can grow in marine media, we inoculated strains from clades α and γ into the following culture media: Volvic mineral water (Danone) enriched with a sterile (boiled) barley grain; 100% Artificial Sea Water (ASW; CCAP recipe); 50% ASW; and 25% ASW. 12 days after the initial inoculations 1 ml of each strain's culture was inoculated as follows: 100% ASW into 50% ASW and vice versa, to test whether viable forms of each strain persisted in 100% ASW even when they could not be seen, and in the second case, to test whether strains were growing (or at least moving around) in 50% ASW had acclimatized to the degree to which they could persist in 100% ASW. Observations reported in Table 2 were made from these experimental cultures and others of the same strains examined over the previous 18 months. Cultures used for these experiments are marked with open circles in Fig. 1. No cultures from clade β were used as they are all dead.

## Authors' contributions

DB coordinated the study, generated the dataset for group α, supervised the generation of datasets for β and γ, and was the main drafting author. DB, TAR, and TCS collected samples. LM and VM generated the datasets and executed preliminary analyses for groups β and γ, respectively. TAR contributed scientific discussion, helped analyze the raw datasets, generated the phylogenies, created the figures, and assisted with the MS. TC-S gave general advice and inspiration and helped with scientific evaluation and writing.
